# Free breathing VMAT versus deep inspiration breath‐hold 3D conformal radiation therapy for early stage left‐sided breast cancer

**DOI:** 10.1002/acm2.13208

**Published:** 2021-02-27

**Authors:** Christer A Jensen, Marit Funderud, Christoffer Lervåg

**Affiliations:** ^1^ Department of Medicine and Healthcare Møre & Romsdal Hospital Trust Ålesund Hospital Ålesund Norway; ^2^ Department of Health Sciences in Ålesund Faculty of Medicine and Health Sciences Norwegian University of Science and Technology (NTNU) Ålesund Norway; ^3^ Department of Oncology Møre & Romsdal Hospital Trust Ålesund Hospital Ålesund Norway

**Keywords:** deep inspiration breath‐hold, free breathing, VMAT, volumetric modulated arc therapy

## Abstract

The purpose of the *in silico* study was to compare free breathing volumetric modulated arc therapy (VMAT) to standard deep inspiration breath‐hold (DIBH) three‐dimensional conformal radiotherapy (3DCRT) and determine whether the former is a viable option for elderly patients with left‐sided early stage breast cancer. Data from 22 patients with early‐stage left breast carcinoma requiring breast‐only radiation therapy were used for this planning study. The robustness of VMAT plans when using the free breathing method was compared to that of standard 3DCRT plans using the DIBH method. The endpoints for evaluation were the target dose coverage as well as doses to the organs‐at‐risk. The free breathing VMAT plans produced a significantly higher mean dose to the heart and right breast than the DIBH‐3DCRT plans. Free breathing VMAT plans resulted in significantly better target coverage than did 3DCRT using DIBH. The external volume that received more than 40 Gy was significantly smaller in the VMAT plans. Free breathing VMAT is a viable alternative to DIBH 3DCRT in elderly patients with a limited life expectancy and in subjects who are unable to perform DIBH. The choice of treatment should be individualized, and all relevant risks ought to be considered.

## 1 INTRODUCTION

Irradiation of the breast after breast‐conserving surgery in patients with early‐stage breast cancer is the current standard of care, as RT has been shown to improve local control and overall survival.^1^ However, radiation‐induced heart diseases and cardiovascular events are well‐documented adverse events in patients with left‐sided breast cancer (LSBC).^2^, ^3^, ^4^ External beam RT for LSBC also delivers a radiation dose to the heart and lung; however, the deep inspiration breath‐hold (DIBH) technique enables the reduction of doses to these organs while maintaining the prescribed dose to the breast.^5^, ^6^, ^7^ Deep inspiration raises the chest wall and expands the volume of the lungs, thereby pushing the heart away from the chest wall and increasing the distance between the target and heart. This method is well established, and several groups have previously reported the benefit of this technique compared to free breathing (FB) RT.^5^, ^8^, ^9^ However, the UK HeartSpare Study showed that the median RT treatment session when using DIBH is 22 min, and this longer time can increase both financial and workforce burdens at high‐activity facilities.^10^ Longer appointments may also lead to the reduced availability of RT.

Volumetric modulated arc therapy (VMAT) is a more recent RT technique that has been investigated in patients with breast cancer.^11^, ^12^, ^13^ VMAT has several advantages over three‐dimensional conformal radiotherapy (3DCRT), including inverse optimization with respect to clinical goals, quick treatment delivery, more homogenous target doses, more conformal dose distributions, and tailored doses to the organs‐at‐risk (OARs). VMAT may therefore improve target coverage and robustness compared to 3DCRT when treating LSBC while lowering doses to the OARs.^11^, ^12^, ^14^, ^15^ Hence, VMAT has increasingly been used to treat patients with breast cancer. In a previous study, it was shown that a VMAT treatment plan could be produced in only a few hours and that the beam‐on time for locoregional breast cancer was three minutes with two arcs.^15^

Radiotherapy is associated with late cardiovascular complications when used to treat LSBC, and cardiovascular‐related mortality in these patients has been found to be significantly higher after 15 yr of follow‐up than it is for patients with right‐sided breast cancer.^3^ One study found that the 10‐year cumulative incidences of major coronary events (such as myocardial infarction, coronary revascularization, and death from ischemic heart disease) in patients with left versus right‐sided tumors were 5.5% and 4.5%, respectively.^4^ Radiotherapy has also been shown to cause secondary malignancies (SM)^16^; the risk of developing a secondary primary cancer of the breast or of another organ after treatment is reported to be higher for women with breast cancer.^16^, ^17^ Of note, elderly patients with short life expectancies might not require RT regimens that use DIBH owing to the small 10‐year cumulative risk of major coronary events, as data show that 10–20 yr generally pass before any heart damage manifests.^18^ Moreover, their risk of developing secondary malignancies during their remaining lifespans is low.^18^

Patients also sometimes find it difficult to perform the DIBH technique, and in‐house experience shows that some patients tend to flex their muscles in the shoulder region or arch their spine to reach the gating amplitude. Additionally, some older patients may struggle to hold their breaths for 20–30 s, which appears to be the general requirement in many clinics. In‐house data have also shown that patients have very different breathing curves throughout their treatment sessions, which may be due to anxiety or illness during some treatment sessions.^19^ These patients receive FB‐3DCRT, which increases the risk of a higher dose to the heart or of exceeding dose guidelines to other OARs. In‐house data show that minimizing the mean dose to the heart is usually preferred, especially in the presence of additional risk factors.

The aim of the study was to compare *in silico* FB‐VMAT planning to DIBH‐3DCRT planning in patients with early‐stage LSBC with respect to target dose coverage and doses to the OARs. It was evaluated whether FB‐VMAT was a suitable technique for elderly patients with breast cancer who had a limited life expectancy. To our knowledge, this is the first study comparing FB‐VMAT with DIBH‐3DCRT.

## 2 MATERIALS AND METHODS

### 2.1 Patient selection and training

The patient population was previously described in detail, all patients were asked for written consent to participate in the Regional Ethics Committee approved protocol.^20^ Briefly, the study included 22 patients referred to Ålesund Hospital with stage pT1–T2N0M0 left breast carcinoma or ductal carcinoma in situ requiring RT, median age 58 (45–74) years. There were no limitations in terms of age or comorbidities. Patients underwent two computed tomography (CT) scans, one with FB for VMAT and the other with DIBH for 3DCRT. The CT scanner was a 16 slice multi‐detector MX8000 Brilliance IDT (Philips Medical Systems, Eindhoven, Netherlands), and images were obtained at a slice thickness of 3 mm. The images were transferred to the Oncentra version 3.4 (Elekta, Crawley, UK) and RayStation version 4.99.1.3 (RaySearch Laboratories AB, Stockholm, Sweden) treatment planning systems (TPS).

### 2.2 Delineation of the target and OARs

A radiation oncologist delineated the clinical target volume (CTV), heart, left anterior descending artery (LAD), and left ventricle (LV), whereas radiation therapists or physicists delineated the lungs, spinal canal, right breast, and external contour. The CTV included only the left breast. The heart was delineated, as well as LV and LAD according to published international guidelines,^21^ whereas the planning target volume (PTV) was derived from the CTV with 10, 5, and 5 mm extensions in the superior‐inferior, anterior‐posterior, and left‐right directions, respectively. The first 5 mm inside the external contour was excluded from both the CTV and PTV.

### 2.3 DIBH‐3DCRT treatment planning

The radiation therapists produced a DIBH‐3DCRT treatment plan in accordance with national guidelines based on an in‐house protocol. 6 MV opposing mono‐isocentric tangential conformal photon beams with low‐weight 6 or 15 MV segments were used to achieve dose homogeneity. The clinical goal for target coverage was a minimum of 95% of the prescribed dose to the CTV. The national guidelines at the time of inclusion stated that the mean dose to the heart should be under 2 Gy, that less than 15% of the left lung should receive more than 20 Gy, and that the mean dose to the CTV should be 50 Gy in 25 fractions. No national guidelines regarding doses to the LAD, LV, spinal canal, or right lung or breast were available at the time of the study, so these were planned according to an in‐house protocol based on the “as low as reasonably achievable” principle. The treatment machine used for modeling was an Elekta Synergy with a 10 mm multi leaf collimator (MLC). The treatment plans were calculated with the “Collapsed Cone” algorithm in Oncentra.

### 2.4 FB‐VMAT treatment planning

The FB‐VMAT treatment plans were generated in RayStation using an Elekta VersaHD treatment machine with a 5 mm MLC for modeling. A set of optimization objectives were established for each VMAT plan to achieve approximately the same target coverage (PTV D_98%_ [Gy]) and heart sparing (mean heart dose) as the original 3D conformal plan (Table 1). To achieve the prescribed dose, two partial 6 MV photon arcs with arc lengths of 240° were used, where the start/stop angles were 179°/280° and the collimator angles were 355°/5°. The maximum delivery time was set to 90 s for each arc.

**Table 1:** Median clinical objectives used for optimization using RayStation.

Structure	Goals	Weight
PTV	98% of volume receive minimum 91.5% of prescribed dose	350
PTV	2% of volume receive maximum 106% of prescribed dose	100
CTV	Uniform dose, prescribed dose	50
CTV	98% of volume receive minimum 94% of prescribed dose	100
Heart	Maximum EUD 2.0 Gy	5
Left lung	Maximum EUD 7.0 Gy	5
Right lung	Maximum EUD 1.0 Gy	5
Lung union	Maximum EUD 4.0 Gy	10
Right breast	Maximum EUD 3.0 Gy	5
External	Maximum dose 104% of prescribed dose	175

Abbreviations: CTV, clinical target volume; PTV, planning target volume.

**Fig. 1:**
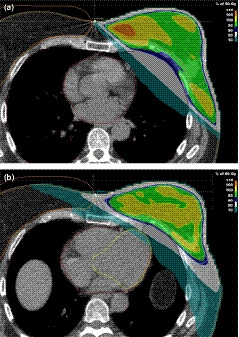
Three‐dimensional conformal plan with tangents placed to encompass the left breast while sparing heart and lung (a), and volumetric modulated arc therapy plan optimized to objectives (b).

The robust optimization feature in RayStation uses a minmax optimization, where a plan is optimized in multiple geometries; the worst (maximum) objective value is used in the objective function. The VMAT optimization was set to be robust with respect to the distal tangent field edge due to breast movement and possible volume changes. The plan was optimized with isocenter offsets applied (in the specified directions mentioned above) to the PTV, which defined the volume for which the plan would be most robust.^22^, ^23^ The optimization objective using the robustness feature had a 10 mm margin in the left and anterior directions. It was assumed that coverage in the other directions would be achieved using the standard PTV margins. The optimized plan was calculated in five different scenarios. Figure 1 shows the typical isodose distributions for each method for the same patient.

### 2.5 Statistics

A two‐tailed Wilcoxon signed‐rank test for the statistical analysis of each evaluated parameter was used, and the test was considered significant if the *P*‐value was <0.05.^24^ SPSS version 23 (IBM Corp., Armonk, NY, USA) was used to perform the calculations.

## 3 RESULTS

**Fig. 2:**
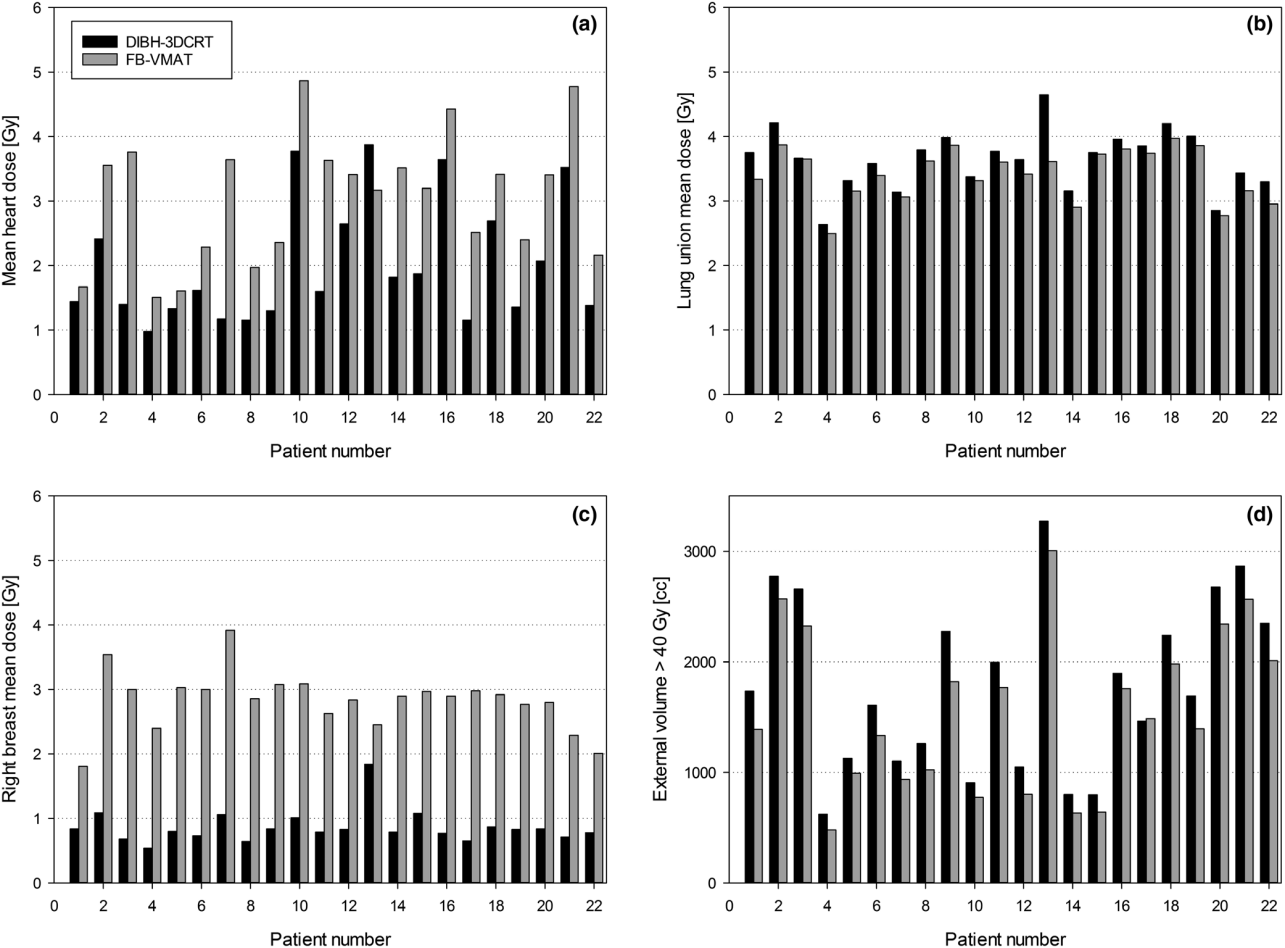
Dosimetric comparisons between the OARs; (a) mean heart dose [Gy], (b) lung union mean dose [Gy], (c) right breast mean dose [Gy] and (d) external volume receiving more than 40 Gy. Black bars DIBH‐3DCRT, gray bars FB‐VMAT.

Treatment planning statistics for all the included patients are shown in Table 2, whereas the mean doses to the selected OARs are shown in Figure 2.

**Table 2:** Dosimetric comparison between DIBH‐3DCRT and FB‐VMAT treatment plans.

Volume	DIBH‐3DCRT	FB‐VMAT
Heart		
Mean [Gy]	2.01 ± 0.94	2.47 ± 0.75*
V_25 Gy_ [%]	0.98 ± 1.38	2.76 ± 3.73
LAD		
Mean [Gy]	11.63 ± 8.12	13.15 ± 8.40
D_2cc_ [Gy]	29.61 ± 17.64	26.90 ± 14.51
Left ventricle		
Mean [Gy]	2.73 ± 1.56	3.18 ± 0.96
V_5 Gy_ [%]	9.55 ± 15.82	9.75 ± 6.70
Left lung		
Mean [Gy]	7.30 ± 0.88	6.23 ± 0.65*
V_20 Gy_ [%]	13.35 ± 1.86	8.72 ± 1.78*
Right lung		
Mean [Gy]	0.54 ± 0.14	0.93 ± 0.19*
V_20 Gy_ [%]	0.36 ± 1.71	0.00 ± 0.00
Lung union		
Mean [Gy]	3.64 ± 0.47	3.32 ± 0.34*
V_5 Gy_ [%]	12.15 ± 1.94	13.16 ± 1.93*
Right breast		
Mean [Gy]	0.86 ± 0.26	2.87 ± 0.54*
PTV		
D_98%_ [Gy]	45.92 ± 0.73	45.95 ± 0.74
V_47.5 Gy_ [%]	93.28 ± 1.79	94.77 ± 1.44*
D_2%_ [Gy]	52.71 ± 0.65	51.17 ± 0.24*
Median [Gy]	49.82 ± 0.12	49.97 ± 0.04*
CTV		
D_98%_ [Gy]	47.11 ± 0.45	48.39 ± 0.38*
V_47.5 Gy_ [%]	97.10 ± 0.85	99.36 ± 0.46*
Mean [Gy]	49.98 ± 0.08	50.00 ± 0.03*
Median [Gy]	49.96 ± 0.05	50.04 ± 0.03*
External CT		
D_2cc_ [Gy]	53.78 ± 0.99	52.97 ± 0.85*
V_40 Gy_ [%]	1780.62 ± 776.71	1511.58 ± 711.18*

Abbreviations: CTV, clinical target volume; D_2 cc_, maximum dose administered to a 2 cm^3^ volume, V_20 Gy_, organ volume receiving 20 Gy; D_2%_, maximum dose administered to 2% of volume; D_98%_, dose to 98% of the target volume; DIBH‐3DCRT, deep inspiration breath‐hold–three‐dimensional conformal radiotherapy; External CT, computed tomography scan volume; FB‐VMAT, free breathing volumetric modulated arc therapy; LAD, left anterior descending artery; PTV, planning target volume.

* indicates significant differences.

### 3.1 Cardiac doses

FB‐VMAT plans produced a significantly higher mean dose to the heart than did DIBH‐3DCRT plans, although the absolute difference was small; the mean dose increased from 2.01 Gy with DIBH‐3DCRT to 2.49 Gy with FB‐VMAT. Notably, the FB‐VMAT technique increased the dose to the heart when compared with DIBH‐3DCRT in 21 of 22 plans. Differences in the remaining heart parameters were not statistically significant.

### 3.2 Target doses

FB‐VMAT plans generally resulted in significantly improved target doses when compared to the DIBH plans, although the absolute differences were small. The largest difference in the PTV dose coverage was at V_47.5 Gy_ [%], where FB‐VMAT plans were significantly better than DIBH‐3DCRT plans.

### 3.3 General statistics

The FB‐VMAT plans produced significantly lower mean doses to the lung union than did the DIBH‐3DCRT plans; moreover, the volumes that received more than 20 Gy (V_20 Gy_ [%]) to the left lung were smaller when using the FB‐VMAT plans. FB‐VMAT plans generally produced a small increase in the low‐dose lung bath (V_5 Gy_ [%]) and a larger increase in the mean dose to the right breast. The external volume that received more than 40 Gy was significantly larger in the DIBH‐3DCRT plans; 21 of 22 plans were associated with larger volumes receiving a high radiation dose.

## 4 DISCUSSION

The DIBH technique delivers lower doses to the OARs than do FB plans that use the same field configurations.^25^ However, the study found that it was possible to produce comparable doses with FB‐VMAT and DIBH‐3DCRT. The FB‐VMAT plans provided similar sparing of the lungs and LAD, but owing to the intrinsic low‐dose bath of VMAT, the mean doses to the heart and right breast were significantly higher than those when using the DIBH‐3DCRT technique. Nevertheless, the absolute OAR differences were small, and the FB‐VMAT plans were therefore clinically acceptable.

The study found a small but significant difference in the mean heart dose between the two techniques. Darby et al’s model predicted that, for a 50‐year‐old woman with no preexisting cardiac risk factors, a mean heart dose increase from 2.0 Gy to 2.5 Gy would increase the risk of death from ischemic heart disease before the age of 80 yr from 2.2% to 2.3% (the baseline risk is 1.9%).^2^ This dose increase will also heighten her risk of having at least one acute coronary event from 5.1% to 5.3% (the baseline risk is 4.5%) For women with one or more preexisting cardiac risk factors, a mean dose increase to the heart from 2.0 Gy to 2.5 Gy would increase the risk of death from ischemic heart disease before the age of 80 yr from 3.9% to 4.0% (the baseline risk is 3.4%).^2^ These added risks are small for older women with limited life expectancy.

Data from the published literature show that 10–20 years generally pass before any heart damage owing to RT manifests.^18^ Generally, the DIBH technique requires more time to perform in the clinic than does a standard FB technique.^26^ In most cases, this additional time will result in extra costs for the clinic and may reduce the availability of RT. Patients who are unable to perform DIBH receive FB‐3DCRT instead, in which case physicians have to balance between delivering a greater mean dose to the heart versus a smaller dose to the target, within the guidelines. In‐house experience shows that minimizing the dose to the heart is usually preferred, especially in the presence of any additional risk factors.

There are general concerns regarding the potential detrimental long‐term effects of the low radiation doses delivered using the VMAT technique. Radiation pneumonitis and fibrosis are well‐known side effects of radiotherapy; while their incidence in patients receiving conformal RT is low, it may increase if appropriate clinical objectives during VMAT optimization are not applied. Large clinical studies of long‐term cancer survivors have found a higher risk of secondary malignancies in those who received RT; these usually occur near tissues irradiated with intermediate doses (ranging from 2 to 50 Gy) or in tissues that have received full but insufficient doses.^27^, ^28^ Whether low doses under 2 Gy have detrimental effects remains unknown.^29^ Patients diagnosed with cancer also have a higher risk of developing new primary cancers in other organs.^30^ There is no clear consensus regarding the acceptable low doses to the lung, but it is expected that the effect of the low‐dose bath (V_5 Gy_ [%]) to the lungs to be too low to significantly increase the risk of pneumonitis.

Abo‐Madyan et al. calculated the secondary cancer risk in patients with breast cancer who received 3DCRT, intensity‐modulated RT (IMRT), and VMAT using various models.^31^ The cumulative (right breast + ipsilateral lung + right lung) additional absolute risk per 10,000 persons per year per Gy at age 70 years after exposure at age 30 years was estimated to be 29 ± 7% using a linear‐exponential model for 3DCRT. Even if the relative difference was larger for VMAT, the absolute risk was low enough that the added risk would have a negligible impact even 40 years after RT. Abo‐Madyan et al. concluded that the added risk would only be clinically relevant if VMAT is adopted as a routine method for young patients. The choice of the optimal treatment should balance relevant risks such as cardiac complications, other deterministic normal tissue damage, and possible stochastic long‐term effects.^31^

Limited long‐term follow‐up data are available for patients who underwent modern RT techniques such as IMRT and VMAT. Improvement in the conformity of treatment has been suggested to counteract the greater risks associated with large low‐dose volumes.^32^ Chargari et al. investigated the impact of IMRT on the risk of secondary cancers due to the greater volumes of normal tissues receiving low doses of radiation using available clinical data.^32^ They concluded that any increased risk was not as high as theoretical models have predicted in adults, with a possible explanation being that the smaller volumes of normal tissues receiving high doses could offset the increase in the low‐dose bath. While the linear no‐threshold model suggested that IMRT could almost double the risk of secondary cancer, their analysis of the literature showed very little evidence of such an increased risk. It is therefore important to consider the benefit versus cost balance in future research.

Kirova et al. reviewed data from 16,705 breast cancer survivors and found that 709 of them had developed a second cancer after a median follow‐up of 10.5 years.^33^ However, they did not find any significant increase in the risk of cancer outside the irradiation field. Their cohort included patients treated between 1981 and 1997 who received low‐energy cobolt‐60 or megavoltage photons with linear accelerators; the tangential opposing beams with wedges produced by these devices would probably have increased the scattered radiation. Woo et al. found that the use of wedges as a compensation technique in breast cancer led to a 2.35 Gy mean dose to the right breast,^34^ which is the same magnitude as that in the FB‐VMAT plans. Berrington de Gonzalez et al. systematically analyzed cancer registries containing 15 cancer sites that are routinely treated with RT.^35^ Patients who achieved 5‐year survival were followed for a mean of 12 yr, whereupon it was found that 9% developed second solid cancers, approximately 8% of which could be linked to radiotherapy (i.e., RT possibly led to 5 additional cancers per 1000 patients within 15 yr after diagnosis). The authors regarded their results as indicating that a relatively small proportion of secondary cancers is related to RT in adults, whereas most are due to other factors such as lifestyle and genetics.

A low average dose to the right breast is a clinical objective when optimizing FB‐VMAT plans; however, the overall mean dose is significantly higher with these plans. Pan et al. performed a systematic review and meta‐analysis of the risk of secondary malignancies after partial versus whole‐breast RT.^36^ They found that partial breast RT, typically administered intraoperatively, produces a very limited dose to the organs surrounding the target compared to whole‐breast external RT, although they found no significant difference in the incidences of secondary non‐breast or right breast cancer between patients receiving partial versus whole‐breast irradiation.

The good VMAT target coverage in the study was not at the expense of a high‐dose bath to healthy tissue. Reidunsdatter et al. found that the V_40 Gy_ was a significant predictor of increased fatigue during RT.^37^ However, the VMAT plans in the study were more conformal than the 3DCRT plans were; the latter was associated with a 17.8% larger tissue volume receiving a high dose (i.e., above 40 Gy) inside the external contour compared to the former. Other groups also discovered significant correlations between the irradiated high‐dose volume (i.e., the tissue encompassed by the 50% isodose) and the intensity of fatigue after RT.^38^, ^39^, ^40^ Therefore, the lower high‐dose volume in the VMAT plans may cause less fatigue in patients with LSBC.

Several groups have attempted to identify simple predictors of cardiac sparing during DIBH for patients with LSBC, but no clear factors have emerged. Age, amplitude, body mass index, and smoking status have been investigated, yet only body mass index appears to be an indicator of when DIBH would be preferred.^41^, ^42^, ^43^, ^44^ Jacobson et al. reported that older patients are generally not as eligible for DIBH as are younger patients.^43^ Additionally, some older patients with dementia may struggle to hold their breath for 20 s and may therefore require more breath‐holds; this can prolong treatment times and possibly increase intrafractional errors.^45^ As the treatment time increases, baseline drift from the setup position has also been shown to increase.^46^ On average, well‐optimized VMAT plans are more robust than 3DCRT counterparts when localization errors during treatment are accounted for.^15^ Older patients with limited life expectancies would therefore be good candidates for FB‐VMAT.

A limitation of the study was that the results were based on estimating the dose at the time of the planning CT scan; patient contours and the inhaled volumes can differ during the actual radiotherapy course. Variations in how the patients perform their breath‐hold during the treatment sessions as well as set‐up variability were not accounted for. This may lead to altered doses to the OARs, especially the heart and the LAD; however, a previous study found that VMAT plans are durable against localizations errors.^15^ Kügele et al. found that 10% of tangential treatments in DIBH had a cumulative probability of having intrafractional DIBH isocenter reproducibility greater or equal to 3.2/3.1/2.1 mm in the lat, long and vert directions, respectively^47^. Another limitation is that the original DIBH‐3DCRT plans were made using another TPS and then imported into RayStation for comparison with FB‐VMAT. Doses between TPSs were calculated for three patients and only minor low‐dose differences were found; the larges absolute difference was less than 0.1 Gy in the mean dose to the contralateral breast. Dissimilar MLC widths may be a potential limitation, although Height et al. found no significant dosimetric differences to support an advantage of 5 mm over 10 mm leaf widths.^48^

In recent years, VMAT has been increasingly used to treat patients with breast cancer; however, there are still no long‐term data, and any cost‐to‐benefit relationship must therefore be modeled. A low‐dose bath has been predicted to increase the risk of secondary cancers; however, no clinical studies to date have shown as high a risk as the theoretical models predicted.^32^ The increased dose to surrounding tissue could possibly be counterbalanced by the improved target coverage, increased target homogeneity, and smaller volume receiving high doses in the VMAT plans.

## 5 CONCLUSIONS

This study shows that FB‐VMAT is a feasible alternative to DIBH‐3DCRT. It is imperative to use well‐founded clinical objectives during optimization to avoid low‐dose bathing of the OARs as much as possible. Further studies should evaluate the clinical outcomes and long‐term risks, and it is necessary to balance the risk of low‐dose exposure and clinical efficacy. The choice of treatment should take into account all associated risks and should ideally be individualized to each patient’s need. FB‐VMAT is also a viable option for elderly patients with LSBC who have limited life expectancies as well as for patients who are ineligible or unable to undergo RT using the DIBH technique.

## CONFLICT OF INTEREST

The authors report no conflicts of interest. The authors alone are responsible for the content and writing of the paper.

## References

[1] Veronesi U , Cascinelli N , Mariani L , et al, Twenty‐year follow‐up of a randomized study comparing breast‐conserving surgery with radical mastectomy for early breast cancer. New Eng J Med. 2002;347:1227–1232. 12393819 10.1056/NEJMoa020989

[2] Darby SC , Ewertz M , McGale P , et al, Risk of ischemic heart disease in women after radiotherapy for breast cancer. New Eng J Med. 2013;368:987–998. 23484825 10.1056/NEJMoa1209825

[3] Henson KE , McGale P , Taylor C , et al, Radiation‐related mortality from heart disease and lung cancer more than 20 years after radiotherapy for breast cancer. Br J Cancer. 2013;108:179–182. 23257897 10.1038/bjc.2012.575 PMC3553540

[4] Boero IJ , Paravati AJ , Triplett DP , et al, Modern radiation therapy and cardiac outcomes in breast cancer. Int J Radiat Oncol Biol Phys. 2016;94:700–708. 26972642 10.1016/j.ijrobp.2015.12.018

[5] Nissen HD , Appelt AL . Improved heart, lung and target dose with deep inspiration breath hold in a large clinical series of breast cancer patients. Radiother Oncol. 2013;106:28–32. 23199652 10.1016/j.radonc.2012.10.016

[6] Swanson T , Grills IS , Ye H , et al, Six‐year experience routinely using moderate deep inspiration breath‐hold for the reduction of cardiac dose in left‐sided breast irradiation for patients with early‐stage or locally advanced breast cancer. Am J Clin Oncol. 2013;36:24–30. 22270108 10.1097/COC.0b013e31823fe481 PMC3375337

[7] Bartlett FR , Colgan RM , Carr K , et al, The UK HeartSpare Study: randomised evaluation of voluntary deep‐inspiratory breath‐hold in women undergoing breast radiotherapy. Radiother Oncol. 2013;108:242–247. 23726115 10.1016/j.radonc.2013.04.021

[8] Korreman SS , Pedersen AN , Nøttrup TJ , et al, Breathing adapted radiotherapy for breast cancer: comparison of free breathing gating with the breath‐hold technique. Radiother Oncol. 2005;76:311–318. 16153728 10.1016/j.radonc.2005.07.009

[9] Vikström J , Hjelstuen MHB , Mjaaland I , et al, Cardiac and pulmonary dose reduction for tangentially irradiated breast cancer, utilizing deep inspiration breath‐hold with audio‐visual guidance, without compromising target coverage. Acta Oncol. 2011;50:42–50. 20843181 10.3109/0284186X.2010.512923

[10] Bartlett FR , Donovan EM , McNair HA , et al, The UK heartspare study (Stage II): multicentre evaluation of a voluntary breath‐hold technique in patients receiving breast radiotherapy. Clin Oncol (R Coll Radiol). 2017;29:e51–e56. 27890346 10.1016/j.clon.2016.11.005

[11] Osman SOS , Hol S , Poortmans PM , et al, Volumetric modulated arc therapy and breath‐hold in image‐guided locoregional left‐sided breast irradiation. Radiother Oncol. 2014;112:17–22. 24825176 10.1016/j.radonc.2014.04.004

[12] Swamy ST , Radha CA , Kathirvel M , et al, Feasibility study of deep inspiration breath‐hold based volumetric modulated arc therapy for locally advanced left sided breast cancer patients. Asian Pac J Cancer Prev. 2014;15:9033–9038. 25374248 10.7314/apjcp.2014.15.20.9033

[13] Boman E , Rossi M , Haltamo M , et al, A new split arc VMAT technique for lymph node positive breast cancer. Physica Med. 2016;32:1428–1436. 10.1016/j.ejmp.2016.10.012 28029580

[14] Bodez V , Duqueyroix F , Fourvel M , et al, Conformal segmented mono‐isocentric radiotherapy vs. volumetric modulated arctherapy in senology. Physica Med. 2016;32:375.

[15] Jensen CA , Roa AMA , Johansen M , et al, Robustness of VMAT and 3DCRT plans toward setup errors in radiation therapy of locally advanced left‐sided breast cancer with DIBH. Phys Med. 2018;45:12–18. 29472076 10.1016/j.ejmp.2017.11.019

[16] Burt LM , Ying J , Poppe MM , et al, Risk of secondary malignancies after radiation therapy for breast cancer: comprehensive results. The Breast. 2017;35:122–129. 28719811 10.1016/j.breast.2017.07.004

[17] Marcu LG , Santos A , Bezak E . Risk of second primary cancer after breast cancer treatment. Eur J Cancer Care (Engl). 2014;23:51–64. 23947545 10.1111/ecc.12109

[18] Sardar P , Kundu A , Chatterjee S , et al, Long‐term cardiovascular mortality after radiotherapy for breast cancer: a systematic review and meta‐analysis. Clin Cardiol. 2017;40:73–81. 28244595 10.1002/clc.22631 PMC6490535

[19] Jensen C , Fallmyr I , Skottner N . Development of a novel respiratory gating system for tangential breast irradiation. Radiat Oncol J. 2012;103(Suppl 1):552.

[20] Jensen CA , Skottner N , Frengen J , et al, Development of a deep inspiration breath‐hold system for radiotherapy utilizing a laser distance measurer. J Appl Clin Med Phys. 2017;18:260–264. 28291926 10.1002/acm2.12011 PMC5689898

[21] Feng M , Moran JM , Koelling T , et al, Development and validation of a heart atlas to study cardiac exposure to radiation following treatment for breast cancer. Int J Radiat Oncol Biol Phys. 2011;79:10–18. 20421148 10.1016/j.ijrobp.2009.10.058 PMC2937165

[22] Fredriksson A , Forsgren A , Hardemark B . Minimax optimization for handling range and setup uncertainties in proton therapy. Med Phys. 2011;38:1672–1684. 21520880 10.1118/1.3556559

[23] Fredriksson A , Bokrantz R . A critical evaluation of worst case optimization methods for robust intensity‐modulated proton therapy planning. Med Phys. 2014;41:81701. 10.1118/1.4883837 25086511

[24] Jensen CA , Abramova T , Frengen J , et al, Monitoring deep inspiration breath hold for left‐sided localized breast cancer radiotherapy with an in‐house developed laser distance meter system. J Appl Clin Med Phys. 2017;18:117–123. 28755403 10.1002/acm2.12137 PMC5875834

[25] Guckenberger M , Kavanagh A , Webb S , et al, A novel respiratory motion compensation strategy combining gated beam delivery and mean target position concept –a compromise between small safety margins and long duty cycles. Radiother Oncol. 2011;98:317–322. 21354640 10.1016/j.radonc.2011.01.008

[26] Suit H , Goldberg S , Niemierko A , et al, Secondary carcinogenesis in patients treated with radiation: a review of data on radiation‐induced cancers in human, non‐human primate, canine and rodent subjects. Radiat Res. 2007;167:12–42. 17214511 10.1667/RR0527.1

[27] Berrington de Gonzalez A , Gilbert E , Curtis R , et al, Second solid cancers after radiation therapy: a systematic review of the epidemiologic studies of the radiation dose‐response relationship. Int J Radiat Oncol Biol Phys. 2013;86:224–233. 23102695 10.1016/j.ijrobp.2012.09.001 PMC3816386

[28] Little MP , Wakeford R , Tawn EJ , et al, Risks associated with low doses and low dose rates of ionizing radiation: why linearity may be (Almost) the best we can do. Radiology. 2009;251:6–12. 19332841 10.1148/radiol.2511081686 PMC2663578

[29] Grantzau T , Overgaard J . Risk of second non‐breast cancer among patients treated with and without postoperative radiotherapy for primary breast cancer: a systematic review and meta‐analysis of population‐based studies including 522,739 patients. Radiother Oncol. 2016;121:402–413. 27639892 10.1016/j.radonc.2016.08.017

[30] Kirova YM , De Rycke Y , Gambotti L , et al, Second malignancies after breast cancer: the impact of different treatment modalities. Br J Cancer. 2008;98:870–874. 18268495 10.1038/sj.bjc.6604241 PMC2266852

[31] Woo TCS , Pignol J‐P , Rakovitch E , et al, Body radiation exposure in breast cancer radiotherapy: impact of breast IMRT and virtual wedge compensation techniques. Int J Radiat Oncol Biol Phys. 2006;65:52–58. 16457966 10.1016/j.ijrobp.2005.11.023

[32] de Gonzalez AB , Curtis RE , Kry SF , et al, Proportion of second cancers attributable to radiotherapy treatment in adults: a cohort study in the US SEER cancer registries. Lancet Oncol. 2011;12:353–360. 21454129 10.1016/S1470-2045(11)70061-4 PMC3086738

[33] Abo‐Madyan Y , Aziz MH , Aly MMOM , et al, Second cancer risk after 3D‐CRT, IMRT and VMAT for breast cancer. Radiother Oncol. 2014;110:471–476. 24444525 10.1016/j.radonc.2013.12.002

[34] Reidunsdatter RJ , Rannestad T , Frengen J , et al, Early effects of contemporary breast radiation on health‐related quality of life – predictors of radiotherapy‐related fatigue. Acta Oncol. 2011;50:1175–1182. 21871005 10.3109/0284186X.2011.604345

[35] Albuquerque K , Tell D , Lobo P , et al, Impact of partial versus whole breast radiation therapy on fatigue, perceived stress, quality of life and natural killer cell activity in women with breast cancer. BMC Cancer. 2012;12:1–12. 22708709 10.1186/1471-2407-12-251 PMC3542587

[36] Geinitz H , Zimmermann FB , Stoll P , et al, Fatigue, serum cytokine levels, and blood cell counts during radiotherapy of patients with breast cancer. Int J Radiat Oncol Biol Phys. 2001;51:691–698. 11597810 10.1016/s0360-3016(01)01657-1

[37] Shah S , Kyrillos A , Kuchta K , et al, A single institution retrospective comparison study of locoregional recurrence after accelerated partial breast irradiation using external beam fractionation compared with whole breast irradiation with 8 years of follow‐up. Ann Surg Oncol. 2017;24:2935–2942. 28766205 10.1245/s10434-017-5953-9

[38] Chargari C , Goodman KA , Diallo I , et al, Risk of second cancers in the era of modern radiation therapy: does the risk/benefit analysis overcome theoretical models? Cancer Metastasis Rev. 2016;35:277–288. 26970966 10.1007/s10555-016-9616-2

[39] Pan X‐B , Huang S‐T , Jiang Y‐M , et al, Secondary malignancies after partial versus whole breast irradiation: a systematic review and meta‐analysis. Oncotarget. 2016;7:71951–71959. 27713125 10.18632/oncotarget.12442 PMC5342135

[40] Czeremszyńska B , Drozda S , Górzyński M , et al, Selection of patients with left breast cancer for deep‐inspiration breath‐hold radiotherapy technique: Results of a prospective study. Rep Pract Oncol Radiother. 2017;22:341–348. 28701900 10.1016/j.rpor.2017.05.002 PMC5496478

[41] Mkanna A , Mohamad O , Ramia P , et al, Predictors of cardiac sparing in deep inspiration breath‐hold for patients with left sided breast cancer. Front Oncol. 2018;8:1–6. 30538954 10.3389/fonc.2018.00564 PMC6277631

[42] Jacobson GM , Watson CN , Zhang J , et al, Mean radiation dose to the heart in patients with left breast cancer with and without breath‐hold technique. J Clin Oncol. 2014;32:85.

[43] Ledsom D , Reilly AJ , Probst H . Assessment of deep inspiration breath hold (DIBH) amplitude and reduction in cardiac dose in left breast cancer patients. Radiography. 2018;24:98–103. 29605120 10.1016/j.radi.2017.11.005

[44] Kapanen M , Laaksomaa M , Pehkonen J , et al, Effects of multiple breath hold reproducibility on treatment localization and dosimetric accuracy in radiotherapy of left‐sided breast cancer with voluntary deep inspiration breath hold technique. Med Dosim. 2017;42:177–184. 28526193 10.1016/j.meddos.2017.02.004

[45] Jensen CA , Acosta Roa AM , Lund J‐Å , et al, Intrafractional baseline drift during free breathing breast cancer radiation therapy. Acta Oncol. 2017;56:867–873. 28464748 10.1080/0284186X.2017.1288924

[46] Height FJ , Kron T , Willis D , et al, Impact of MLC leaf width on the quality of the dose distribution in partial breast irradiation. Med Dosim. 2012;37:37–41. 21741821 10.1016/j.meddos.2010.12.011

